# Performance Enhancement of Biopolyester Blends by Reactive Compatibilization with Maleic Anhydride-Grafted Poly(butylene succinate-*co*-adipate)

**DOI:** 10.3390/polym16162325

**Published:** 2024-08-16

**Authors:** Kerly Samaniego-Aguilar, Estefania Sanchez-Safont, Ignacio Pisa-Ripoll, Sergio Torres-Giner, Yaiza Flores, Jose M. Lagaron, Luis Cabedo, Jose Gamez-Perez

**Affiliations:** 1 Polymers and Advanced Materials Group (PIMA), Universitat Jaume I, Av. Sos Baynat s/n, 12071 Castelló, Spain; samanieg@uji.es (K.S.-A.); esafont@uji.es (E.S.-S.); pisa@uji.es (I.P.-R.); lcabedo@uji.es (L.C.); 2 Institute of Food Engineering—FoodUPV, Polytechnic University of Valencia (UPV), Camino de Vera s/n, 46022 Valencia, Spain; yflofer@upv.edu.es; 3 Novel Materials and Nanotechnology Group, Institute of Agrochemistry and Food Technology (IATA), Spanish National Research Council (CSIC), Calle Catedrático Agustín Escardino Benlloch 7, 46980 Paterna, Spain; lagaron@iata.csic.es

**Keywords:** poly(hydroxybutyrate-co-hydroxyvalerate), poly(butylene succinate-co-butylene adipate), polymer blend, maleic anhydride grafting, reactive extrusion

## Abstract

Poly(3-hydroxybutyrate-*co*-3-hydroxyvalerate) (PHBV) is a very promising biodegradable copolyester of high interest in food packaging. Its inherent brittleness and narrow processing window make it necessary to blend it with flexible biopolyesters, such as poly(butylene succinate-*co*-adipate) (PBSA). However, the resultant biopolyester blends are thermodynamically immiscible, which impairs their performance and limits their applications. This study is the first to explore the use of poly(butylene succinate-*co*-adipate) grafted with maleic anhydride (PBS-*g*-MAH) as a novel reactive additive to compatibilize PHBV/PBSA blends. The compatibilizer was prepared by a reactive melt-mixing process of PBSA and maleic anhydride (MAH) using dicumyl peroxide (DCP) as an organic radical initiator, achieving a grafting degree (G_d_) of 5.4%. Biopolyester blend films were thereafter prepared via cast extrusion and their morphological, thermal, mechanical, and barrier properties were characterized. Compatibilization by PBSA-*g*-MAH was confirmed by observing an improved phase interaction and lower dispersed domain sizes in the blends with 15 wt% PBSA. These compatibilized PHBV/PBSA blends were thermally stable up to 285 °C, showed enhanced ductility and toughness, as well as providing an improved barrier against water and limonene vapors and oxygen. These findings suggest that the use of MAH-grafted biopolyesters can represent an effective strategy to improve the properties of biopolyester blends and open up new opportunities for the application of PHBV-based formulations for food packaging.

## 1. Introduction

Polyhydroxyalkanoates (PHAs) are a family of thermoplastic polyesters of bacterial origin that act as carbon and energy stores when the presence of other nutrients, such as oxygen, phosphorus, or nitrogen, is limited. These polymers can accumulate in high concentrations inside the cell without causing any changes. PHAs are composed of different monomeric units of (R)-hydroxy fatty acids. The classification of PHAs varies according to the number of carbon atoms in these units, dividing them into short-chain length (between 3 and 5 carbon atoms) and medium-chain length (between 6 and 18 carbon atoms) [1,2,3]. PHAs are non-toxic fully biodegradable polymers and derived from biological sources, exhibiting properties comparable to those of some conventional plastics. These properties make them a promising group of biopolymers to replace conventional plastics. However, they still show high costs and certain complexities in their processing [4,5].

Within the family of PHAs, the homopolymer poly(3-hydroxybutyrate) (PHB) and its copolymer poly(3-hydroxybutyrate-*co*-3-hydroxyvalerate) (PHBV) currently play the main roles in the biopolymer industry [6]. PHBV copolyesters can have different proportions of 3-hydroxyvalerate (3HV), influencing their final properties accordingly. This biopolyester is semi-crystalline, biodegradable, and exhibits high mechanical resistance and is a good barrier to water vapor and oxygen. However, it presents some drawbacks that limit its use, for instance, a narrow processing window, high production cost, intrinsic brittleness, and low toughness [4,7,8,9]. A common strategy to overcome the weaknesses of PHBV is to blend it with more ductile biopolymers [10]. In this regard, PHBVs have been blended with polylactide (PLA) [11,12], poly(ε-caprolactone) (PCL) [12,13], poly(butylene adipate-*co*-terephthalate) (PBAT) [14,15,16], poly(butylene succinate) (PBS) [8,17], and poly(butylene succinate-*co*-adipate) (PBSA) [18,19], among others. These studies were addressed to improve the performance of PHBV without compromising its biodegradability. 

In the context of biopolymer blends, PBSA is a biodegradable aliphatic copolyester synthesized by the polycondensation reaction of 1,4-butanediol with succinic acid and adipic acid [20]. These monomers can be obtained from natural and renewable resources [21]. PBSA has a remarkable elongation at break and a low glass transition temperature (T_g_) of −45 °C [22], which gives it high flexibility. It also exhibits excellent impact, thermal, and chemical resistance. These properties facilitate its processing using melt techniques, such as extrusion, injection molding, or thermoforming. Furthermore, its low crystallinity and flexibility favor its biodegradation in different environments [18,23,24,25]. Some studies have shown the benefits of blending PHBV with PBSA [7,18,19,26]. However, the absence or low miscibility between the two biopolyesters remains a challenge. Righetti et al. showed this lack of compatibility in a recent study [19], proving that the crystallization of PHBV at higher temperatures excluded PBSA from the crystal domains, hence segregating both phases during solidification. Indeed, other studies have shown that there is no miscibility involved between both polymers [27].

One of the most effective approaches to improve the interfacial adhesion in polymer blends and polymer composites is the use of reactive compatibilizers during melt processing to either chemically modify the surface of the components or introduce chemical bonds among them. The so-called reactive extrusion (REX) is a blending technology that has proven to be highly effective in improving the compatibility between two immiscible polymers. It is based on the use of bi-functional and multi-functional molecules or macromolecules (oligomers and polymers) that can react with the terminal or functional groups of polymers present in the blend. Moreover, REX is considered as a “green” method since it can be performed with the currently available melt-processing equipment, which significantly improves the commercial viability and cost competitiveness of the resulting materials [28,29].

Among the various reactive compatibilizers, the use of maleic anhydride (MAH) grafted onto PHBV has been proven to be an effective strategy in the cases of blends [30,31,32] and composites [17,33,34]. Using this approach, Kennouche et al. [34] demonstrated that the incorporation of MAH-grafted PHBV (PHBV-*g*-MAH) allowed for high compatibilization in composites of PHBV/PBS/halloysite nanotubes. Rodriguez-Uribe et al. [17] also used the same approach for formulations of bio-based PBSA with talc and starch. In another study, Rojas-Lema et al. [33] developed blends of PBS with pistachio shell flour compatibilized with PBS grafted with maleic anhydride (PBS-*g*-MAH). As the procedure to modify the polymer matrix with MAH has proved effective in the aforementioned references, we hypothesize that the same principle could work to compatibilize blends of PBSA with PHBV during melt mixing. Hence, a small fraction of PBSA-g-MAH added to a PHBV/PBSA system during melt mixing would work as a compatibilizer between both polymers, improving the overall performance of their blends.

The objective of the study was to improve the mechanical properties, specifically toughness, and barrier properties against water, limonene vapors, and oxygen of PHBV/PBSA blends through reactive extrusion (REx) using PBSA grafted with maleic anhydride (PBSA-g-MAH). This reactive copolyester was synthesized via a melt-grafting process, initiated by dicumyl peroxide (DCP) as a radical initiator in a mini-mixer. Subsequently, PBSA-g-MAH was incorporated at 3 parts per hundred resin (phr) of the biopolyester blend to compatibilize formulations of PHBV with PBSA at contents of 15 wt% and 30 wt%, using cast extrusion to form films. The biopolyester blend films were then characterized in terms of their morphological, thermal, mechanical, and barrier properties to assess the compatibilizing effect of PBSA-g-MAH on the PHBV/PBSA blends.

## 2. Materials and Methods

### 2.1. Materials

Pellets of PHBV containing 3 %mol 3HV (PHI002) were purchased from Naturplast (Ifs, France), while PBSA (BioPBS FD92PB) [35] was supplied by Mitsubishi Chemical Group (Düsseldorf, Germany). MAH, DCP, D-limonene, and magnesium nitrate (Mg(NO_3_)_2_) were all purchased from Sigma-Aldrich S.A. (Madrid, Spain).

### 2.2. Grafting Procedure

The grafting reaction was carried out in an internal mini-mixer (HAAKE PolyLab^TM^ QC, Thermo-Fisher Scientific, Karlsruhe, Germany) in the presence of DCP as the peroxide initiator based on the procedure established by Phua et al. [36]. To this end, the PBSA pellets were physically premixed with the MAH and DCP powders at contents of 10 and 0.6 phr of PBSA, respectively, and melt-mixed at 110 °C for 7 min. Thus, the resultant mix was purified by refluxing for 4 h in chloroform (Panreac S.A., Barcelona, Spain) and the hot solution was filtered and precipitated into cold methanol (Sigma-Aldrich S.A.). Finally, it was washed with methanol several times to remove any unreacted reagents and dried for 24 h at 50 ± 2 °C under vacuum (J.P. Selecta, S.A., Barcelona, Spain). The samples were kept away from water in a desiccator until further use.

The degree of grafting (G_d_) for PBSA-g-MAH was determined by titration using Equation (1), as described previously by Rojas-Lema et al. [33]. For this, 1 g of PBSA-*g*-MAH was refluxed in 100 mL of chloroform for 1 h. Then, 10 mL of distilled water was added and immediately titrated with 0.025 M of potassium hydroxide (KOH, Sigma-Aldrich S.A.) using phenolphthalein (Fisher Scientific SL, Madrid, Spain).

(1) $$G_{d}\left(\%\right)=\left[\frac{N\cdot \left(V_{1}-V_{0}\right)\cdot 98.06}{1000\cdot W\cdot 2}\right]\times 100$$

where N is the KOH concentration (M); V_0_ and V_1_ represent the KOH volume (mL) for the blank solution and for the titration of PBSA-*g*-MAH, respectively; and W is the sample weight (g). A G_d_ value of 5.4 ± 0.3% was achieved and 98.06 was the MAH molecular weight. 

**Figure 1:**
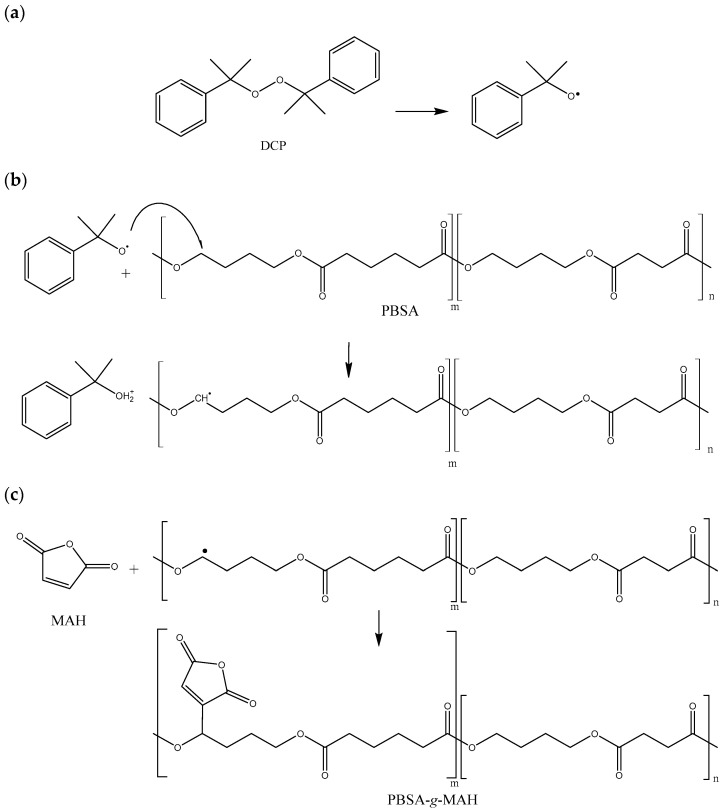
The schema illustrates the steps of the grafting process: (**a**) dicumyl peroxide (DCP) decomposition, (**b**) formation of macroradical, and (**c**) grafting of maleic anhydride (MAH) onto poly(butylene succinate-co-adipate) (PBSA), resulting in poly(butylene succinate-co-adipate) grafted with maleic anhydride (PBSA-g-MAH).

Figure 1 shows the reaction mechanism to obtain PBSA-*g*-MAH from PBSA, MAH, and DCP. This figure is referred to as an example, where the primary radical attack was suffered by the succinate section of the PBS. Statistically, this radical could also attack the adipate fraction of the copolymer, although the overall result would be similar in terms of grafting functional groups that can alter the chemical compatibility of the PBSA. As reported previously [33], the grafting process was induced by the organic radical initiator and based on the formation of macroradicals derived from the hydrogen abstraction of the biopolyester backbone. Briefly, the reaction started with the thermal decomposition of DCP to form primary free radicals (Figure 1a). The DCP radicals give rise to the initiation step, where primary radicals promote hydrogen abstraction from chain transfer reactions on the PBSA backbone, yielding macroradicals (Figure 1b). The PBSA-MAH macroradicals propagate and set off the grafting of MAH onto PBSA(Figure 1c). The reaction continues until the macroradicals undergo hydrogen transfer from another PBSA chain, MAH, or the initiator, or, alternatively, react with other radicals in the system, such as PBSA, MAH, or primary radicals, forming the so-called PBS-*g*-MAH.

### 2.3. Cast-Film Extrusion

Before extrusion, PHBV, PBSA, and PBSA-g-MAH were dried at 60 °C for at least 12 h in an oven (Memmert Basic UFB 500, Memmert GmbH, Schwabach, Germany). The materials were premixed manually and fed into a single-screw extruder equipped with an L/D ratio = 25 (Teach line E20T, Collin, Mintenbeth, Germany). The flat nozzle was coupled to a calendrer to obtain 500 µm thick sheets. The temperature profile in the extruder, from hopper to nozzle, was set at 175/175/165/165/165/165 °C, and the rotation speed was 40 rpm. Table 1 summarizes the film samples prepared, including their nomenclature and composition.

### 2.4. Film Characterization

#### 2.4.1. Morphology

The morphology of PHBV/PBSA blend films was studied by scanning electron microscopy (SEM) using a high-resolution field emission microscope JEOL 7001F (Tokyo, Japan). Prior to SEM observation, samples were cryofractured in liquid nitrogen and coated with a thin layer of platinum by sputtering. The diameter of the droplets corresponding to the dispersed phase was measured using Fiji^®^ software (ImageJ 1.54f) (National Institutes of Health in Bethesda, MD, USA), from carefully selected SEM images magnified at 1500×. For each blend, the average droplet size (d) and the droplet size distribution parameters d_10_, d_50_, and d_90_ were determined, which correspond to the sizes where 10%, 50%, and 90% of the droplets were included, respectively.

SEM micrographs from fractured surfaces of deeply double-edged notched tensile (DDENT) specimens were also analyzed with the purpose to observe the development of plastic deformation (if any) during the fracture process. The chosen ligament length was set at 5 mm and the testing rate for fracture was 10 mm/min.

#### 2.4.2. Thermal Analyses

Thermogravimetric analysis (TGA) was performed using a TGD-STDA Mettler Toledo model TGA/SDTA851e/LF/1600 (Mettler Toledo, Barcelona, Spain) to determine the thermal stability of the PHBV/PBSA blends. The films were heated from 40 °C to 900 °C at a heating rate of 10 °C/min under nitrogen flow. The onset degradation temperature (T_5%_, measured at 5% weight loss) was determined from the weight loss curve. The maximum degradation temperature (T_d_) was determined at the maximum of the derived thermogravimetric analysis (DTG) peak.

Differential scanning calorimetry (DSC) experiments were performed using a DSC instrument (Mettler Toledo, Barcelona, Spain) equipped with an intracooler (Julabo FT900). Before use, the equipment was calibrated with pans using indium as the standard. Samples, weighing between 4 and 6 mg, were subjected to the following 5-step program: first, heating from 20 °C to 190 °C at a rate of 10 °C/min, isotherm at 190 °C for 3 min, cooling to −65 °C at 10 °C/min, isotherm at −65 °C for 3 min, and second heating to 190 °C at 10 °C/min.

The melting temperatures (T_m_) and enthalpies (ΔH_m_) and the crystallization temperatures (T_c_) and enthalpies (ΔH_c_) were, respectively, determined from the second heating and cooling curves. To evaluate the effect of the secondary phase on PHBV crystallization, the crystallinity (X_c_) of each biopolyester was calculated using the following expression:

(2) $$X_{c}\left(\%\right)=\frac{\Delta H_{m}}{{\Delta H}_{m}^{0}\times w}\times 100$$

where ΔH_m_ (J/g) is the melting enthalpy of PHBV or PBSA, ${\Delta H}_{m}^{0}$ is the melting enthalpy of 100% crystalline PHB (146 J/g) [37] or PBSA (135 J/g) [38], and *w* is the polymer weight fraction of PHBV or PBSA in the blend.

#### 2.4.3. Mechanical Characterization

The mechanical properties were evaluated by a tensile test. Dumbbell-shaped specimens were obtained from the films, with one set cut in the machine direction (MD) and the other set cut in the transverse direction (TD). The tests were performed using a universal testing machine (Shimadzu AGS-X 5000N, Kyoto, Japan) equipped with a 500 N load cell, operating at room temperature with a crosshead speed of 10 mm/min. The specimens were tested after a period of 15 days to consider the effect of secondary crystallization on their mechanical performance.

Tear tests were also carried out on MD and TD using the same equipment, in accordance with the UNE-EN ISO 6383-1/200 standard [39], at 200 mm/min until failure. From the corresponding force versus displacement curves, the tear strength was calculated as the average tear force per unit thickness. Samples were tested at 0 and 15 days of aging. All samples were stored in a vacuum desiccator at room temperature until testing.

#### 2.4.4. Permeability Test

The water vapor permeability (WVP) of the films, expressed in kg·m/Pa·s·m^2^, was determined gravimetrically following ASTM E96/E96M [40]. The samples were cut and placed in circular cups (Ø = 3.5 cm) containing 5 mL of distilled water (100% RH). The cups were then placed in desiccators maintained at 25 °C and 53% RH using an oversaturated Mg(NO_3_)_2_ solution. The systems were weighed every hour for 24 h. The water vapor transmission rate (WVTR) of the samples was determined from the slope of the weight loss vs. time curve and corrected for permeant partial pressure to yield permeance. Finally, the permeability value was obtained by correcting for the film thickness. For D-limonene permeability (LP), the procedure was similar to that used for water vapor, with the difference being that 5 mL of D-limonene was placed inside the Payne permeability cups. These cups were stored under the same controlled conditions of 25 °C and 53% RH. In both tests, cups with aluminum films were used as control samples to estimate and subtract the vapor loss through the sealing. Furthermore, films without water and D-limonene were used to correct the mass corresponding to the vapor produced in the film samples during analysis. For the monolayers, permeance was corrected for sample thickness to obtain permeability. All the vapor permeability measurements were performed in triplicate.

Oxygen permeability (OP) was determined by following the ASTM standard method D3985-05 [41]. For this, films of 50 cm^2^ were placed in the Ox-Tran equipment (Model 1/50, Mocon, Minneapolis, MN, USA) at 25 °C and 53% RH. Oxygen permeance was calculated by dividing the oxygen transmission rate (OTR) by the difference in oxygen partial pressure between the two sides of the film and, thereafter, corrected with film thickness to obtain permeability.

## 3. Results

### 3.1. Morphological Characterization 

**Figure 2:**
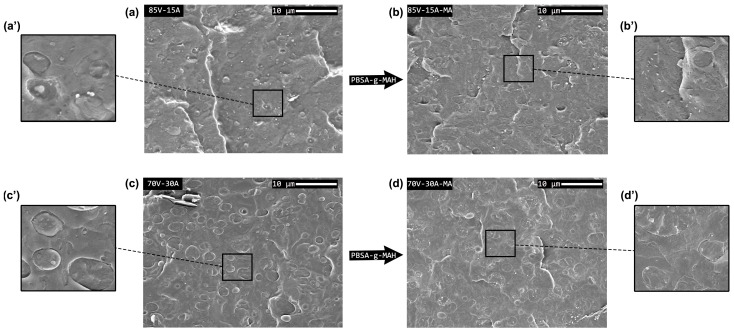
Scanning electron microscopy (SEM) micrographs of the fracture surfaces of (**a**,**a′**) 85V-15A; (**b**,**b′**) 85V-15A-MA; (**c**,**c′**) 70V-30A; (**d**,**d′**) 70V-30A-MA. Images taken at 1000× and 5000×.

The morphology of the PHBV/PBSA blend films with and without PBSA-*g*-MAH was analyzed by SEM. Micrographs of their surface fractures are presented in Figure 2.

The micrographs revealed that all blends exhibited a “drop in matrix” morphology, a characteristic feature of immiscible binary polymer blends. This observation is consistent with other studies involving similar blends [19] as well as in other combinations of PHBV with a more ductile secondary phase, such as PBAT [42] or TPU [9]. More precisely, the micrographs show that the microstructure of the blends consists of domains or droplets of PBSA uniformly distributed throughout the continuous PHBV matrix. In the blends that were processed without PBSA-*g*-MAH, one can also see that the PBSA regions present smooth surfaces with clear edges, indicating poor compatibility and a weak interfacial interaction between the phases. On the other hand, with the addition of PBSA-*g*-MAH to the blends, the edges of the PBSA droplets become indiscernible, indicating an improvement in the interaction between the phases.

Based on the SEM micrographs, particle size measurements were conducted on the blends, and the resulting droplet size distributions (d10, d50, and d90) are summarized in Table 2. In all cases, the droplet size increased with the amount of PBSA in the blend. This behavior aligns with the theory of droplet coalescence, which suggests that, during the mixing process, the dispersed phase can collide and merge, forming larger droplets. Similar observations have been reported in PLA/PCL blends [20,43]. Therefore, the probability of droplet collision further increased with an increase in PBSA content. Moreover, the particle size of the blends exceeded the optimum range to improve the properties of the material, typically between 0.2 and 0.4 µm. Additionally, particle size also determines the ductile-to-brittle transition range of the blend, which is typically in the range of 0.1–1 µm [44,45]. The average droplet sizes in the 85V-15A and 70V-30A blends were 1.47 µm and 2.45 µm, respectively. However, the size of the PBSA droplets remained unchanged upon the addition of PBSA-*g*-MAH to the blends. The particle sizes of the blends (>1 µm) suggest the presence of particles that could act as Griffith flaw initiators, indicating the brittle behavior of the material.

### 3.2. Thermal Characterization

**Figure 3:**
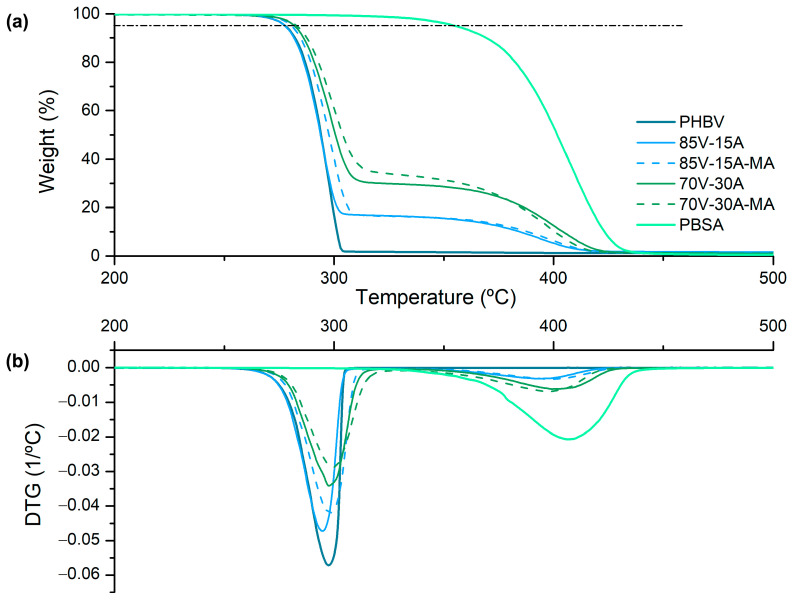
(**a**) Thermogravimetric analysis (TGA) and (**b**) derived thermogravimetric analysis (DTG) curves of neat poly(3-hydroxybutyrate-*co*-3-hydroxyvalerate) (PHBV), poly(butylene succinate-*co*-adipate) (PBSA), and their blends.

TGA was performed to analyze the thermal stability of the PHBV/PBSA blends. Both weight loss and DTG curves are shown in Figure 3. Table 3 includes the onset degradation temperature or temperature to attain a mass loss of 5% (T_5%_), indicated with a dashed line in Figure 3a and degradation temperature of both PHBV (T_d1_) and PBSA (T_d2_) obtained from the maximum DTG peaks. 

The typical thermal degradation of pure biopolyesters occurs in a single step. For PHBV, the T_5%_ (temperature at 5% weight loss) and T_d_ (degradation temperature) values were approximately 280 °C and 300 °C, respectively, consistent with previous findings. [46]. PBSA demonstrated greater thermal stability, beginning degradation at 357 °C and exhibiting a Td value of 407 °C [18]. In the blends, the T_5%_ values were predominantly influenced by PHBV, with a slight shift to higher temperatures as the PBSA content increased, due to the dilution effect of PHBV with the more thermally stable PBSA. Thermograms also displayed two degradation peaks corresponding to the PHBV and PBSA phases [27]. Additionally, the incorporation of PBSA-g-MAH slightly improved the thermal stability of the PHBV/PBSA blends compared to those without compatibilization.

**Figure 4:**
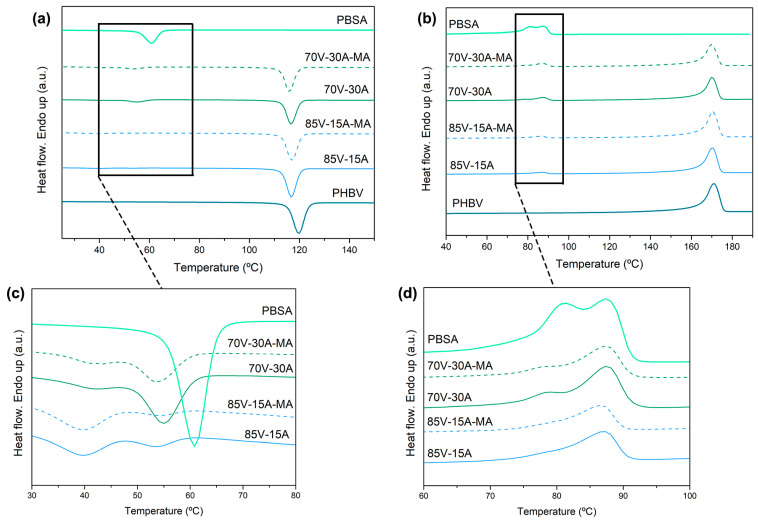
Differential scanning calorimetry (DSC) curves of neat poly(3-hydroxybutyrate-*co*-3-hydroxyvalerate) (PHBV), poly(butylene succinate-*co*-adipate) (PBSA), and their blends: (**a**) cooling curves; (**b**) second heating curves; (**c**) zoomed in image of cooling curves; (**d**) zoomed in image of heating curves.

Regarding the heating/cooling behavior, Figure 4 shows the DSC thermograms of the neat PHBV and PBSA biopolymers and the different blends. The results are summarized in Table 4, where T_C1_, T_m1_, ΔH_c1_, and ΔH_m1_ correspond to the temperatures and enthalpies of crystallization and melting of PHBV, whereas T_C2_, T_m2.1_, T_m2.2_, ΔH_c2_, and ΔH_m2_ correspond to the ones of PBSA.

The thermograms in Figure 4c reveal two distinct crystallization peaks in the cooling curves of the blends, indicating that the crystallization of the two components occurs sequentially. The thermograms in Figure 4c reveal a slight shift toward lower T_c_ values for PHBV (shifting to 116–118 °C) after blending with PBSA [34]. This slight delay in the crystallization of PHBV during cooling can be attributed to the two-phase morphology in the blend, where the domain boundaries interfere and restrict chain mobility. A similar restriction in crystallization growth was observed for PBSA in the blends. 

However, in this case, the biocopolyester developed two crystallization peaks (T_c2.1_ and T_c2.2_) at approximately 55 °C and 40 °C, respectively. This double-crystallization phenomenon has been attributed in linear polyesters to different crystallization kinetics due to spatial restrictions inside the PBSA droplets, which produces populations of different lamellar thicknesses [18,34,47]. This is consistent with the different droplet sizes observed by SEM. In terms of the total crystallinity developed during cooling, both biopolymers exhibited intermediate overall ΔH_c_ values based on their weight fractions in the blends. Furthermore, the addition of PBSA-*g*-MAH did not significantly affect the crystallization behavior of either PHBV or PBSA in the blends, as deduced from the values reported in Table 4.

The heating curves (Figure 4b) showed a single melting peak for PHBV, centered at 171 °C, while two melting peaks were observed for PBSA; the first occurred at 83 °C and the second at 89 °C. The phenomenon of multiple melting peaks in PBSA has been widely studied and can be attributed to recrystallization during the melting process upon heating [26,48,49,50]. For the blend with 30 wt% of PBSA, two peaks were also observed. The blends also exhibited intermediate ΔH_m_ values based on the weight fraction of each biopolyester in the blends and the crystallinity remained nearly constant, reaching X_c_ values in the ranges of 60–63% and 27–31% for PHBV and PBSA, respectively. This result suggests the low interaction of both phase components during melting, which was not affected by the PBSA-*g*-MAH incorporation during blending.

### 3.3. Mechanical Characterization

**Figure 5:**
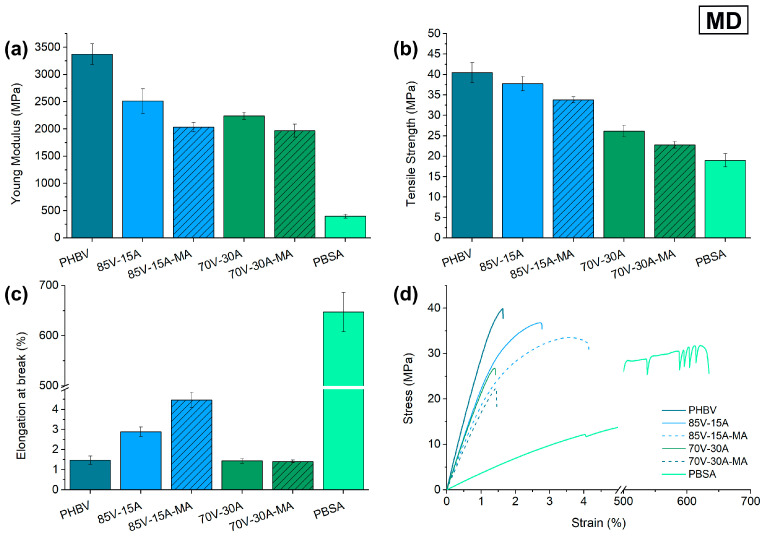
Tensile mechanical properties of neat poly(3-hydroxybutyrate-*co*-3-hydroxyvalerate) (PHBV), poly(butylene succinate-*co*-adipate) (PBSA), and their blends in the machine direction (MD): (**a**) modulus of elasticity; (**b**) tensile strength; (**c**) elongation at break; (**d**) stress–strain curves.

**Figure 6:**
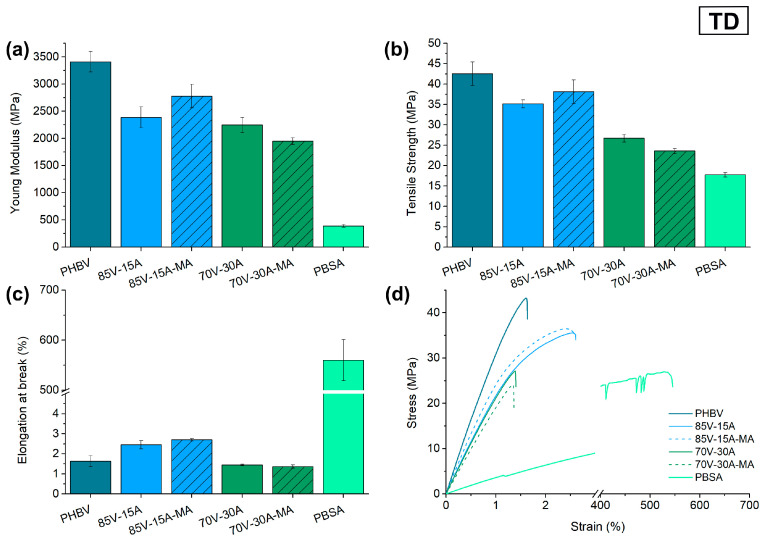
Tensile mechanical properties of neat poly(3-hydroxybutyrate-*co*-3-hydroxyvalerate) (PHBV), poly(butylene succinate-*co*-adipate) (PBSA), and their blends in transverse direction (TD): (**a**) modulus of elasticity; (**b**) tensile strength; (**c**) elongation at break; (**d**) stress–strain curves.

The mechanical characterization of the cast-extruded films of the binary PHBV/PBSA blends, with and without PBSA-*g*-MAH, was studied by tensile and tear tests. As PHBV is known to develop physical aging and secondary crystallization, both of which can affect its mechanical behavior, tests were carried out after 15 days of storage in room conditions [51]. The Young’s Modulus (E), tensile strength (σ_max_), elongation at break (ε_b_), as well as the stress–strain curves for MD and TD are compiled in Figure 5 and Figure 6, respectively. It can be observed that the neat PHBV film shows the properties of a rigid and strong material, with E and σ_max_ values in the ranges of 3000–3500 MPa and 40–50 MPa, respectively. However, it also presents a typical brittle behavior, which is evidenced from its low ductility, that is, low ε_b_ values (<2%). In contrast, PBSA resulted in a flexible and ductile material, with E, σ_max_, and ε_b_ values in the ranges of 200–300 MPa, 15–20 MPa, and 500–700%, respectively. 

The addition of PBSA to the blend decreased the stiffness and strength of PHBV, but increased its ductility. This behavior is expected when adding a more ductile second phase [7,19,52]. Particularly, the E values of the 85V-15A and 70V-30A blend films decreased by approximately 26% and 33%, respectively, with respect to the neat PHBV film in MD. However, it is worth noticing that only when the amount of PBSA was 15%, the ε_b_ value increased, reaching a percentage increase of 93% with respect to the PHBV film. 

When PBSA-*g*-MAH was added to PHBV, there was a slight loss of stiffness and mechanical resistance in MD for both blends when compared to their respective uncompatibilized blend films. However, for the 85V-15A-MA blend film in TD, the E and σ_max_ values increased by 16% and 8%, respectively, with respect to blends without PBSA-*g*-MAH. The ductility also increased with the use of the reactive compatibilizer in both MD and TD, reaching increases in the ε_b_ values of 55% and 8%, respectively. These results suggest that PBSA-*g*-MAH can compatibilize moderate amounts of PBSA in PHBV, which is the case for the 85:15 (*wt*/*wt*) blend. On the other hand, this effect was not so clearly observed in the 70:30 (*wt/wt)* blend [7].

Two factors can influence the tensile behavior of the blends. Compatibility between the phases [53,54,55] and size of the domains [20,44,45]. In the case of the 85:15 blend, it seems that the amount of PBSA-g-MA is enough to promote some compatibilization between PHBV and PBSA, hence showing some improvement in the elongation at break and an overall better performance in tensile strength, balancing MD and TD values. In the case of the 70:30 blend, it was already seen by SEM that the size of the droplets was much larger than in the case of the 85:15 composition. Altogether, it could be reasoned that the compatibilizing effect of PBSA-g-MA added to the 70:30 blend was not enough to promote a shift in the tensile properties, controlled by the domain size of the particles. Looking at the elongation at break of the blends, the effect of the PBSA can be related to the particle size, regardless of the presence of a compatibilizer, presenting an increase in the strain at break in the 85:15 blend and a reduction in the case of the 70:30 blends.

**Figure 7:**
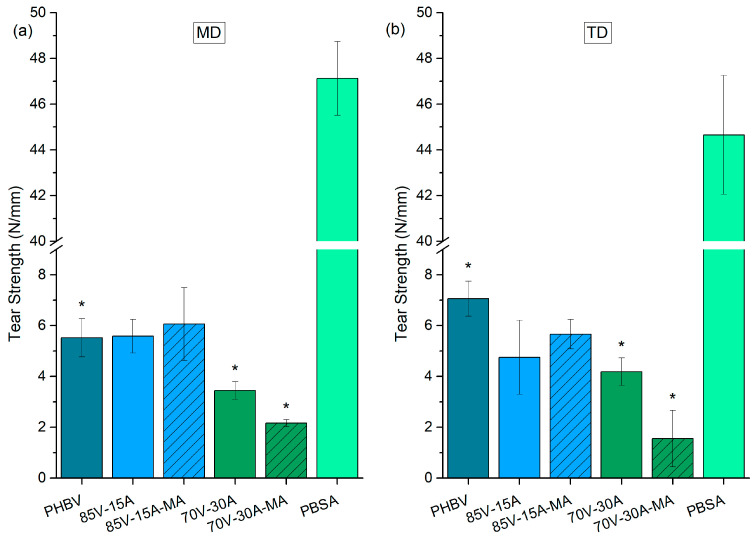
Tear strength in the machine direction (MD) (**a**) and transverse direction (TD) (**b**) for neat poly(3-hydroxybutyrate-*co*-3-hydroxyvalerate) (PHBV), poly(butylene succinate-*co*-adipate) (PBSA), and their blends. * Crack deviation during the test.

**Figure 8:**
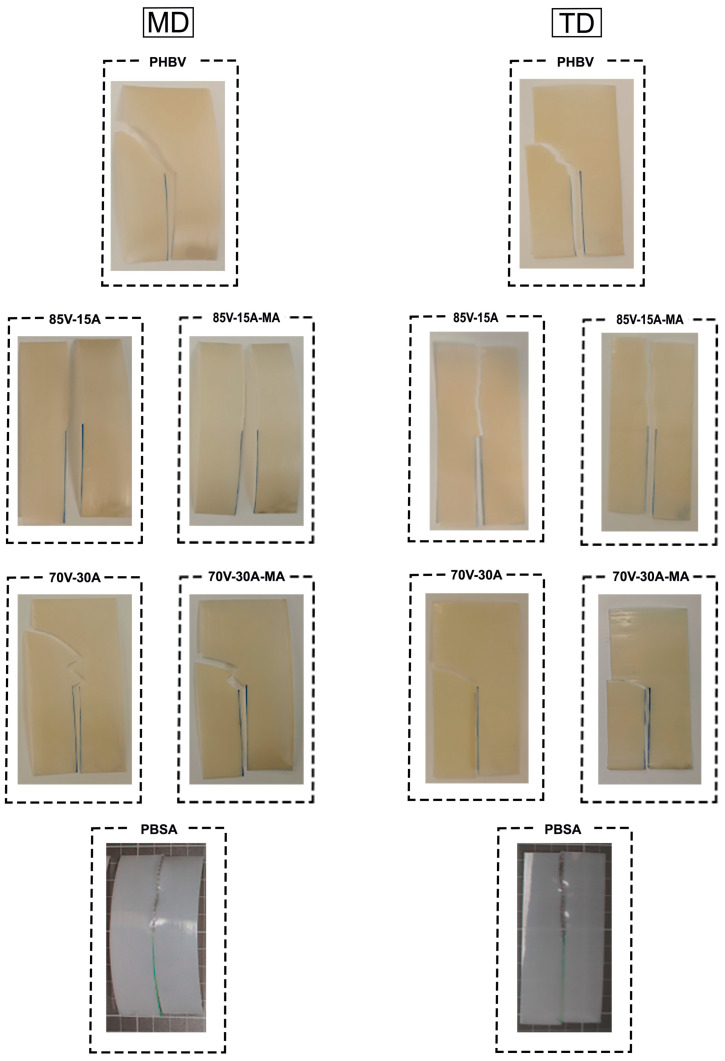
Specimens tested for tear strength in the machine direction (MD) and transverse direction (TD) of neat poly(3-hydroxybutyrate-*co*-3-hydroxyvalerate) (PHBV), poly(butylene succinate-*co*-adipate) (PBSA), and their blends.

Tear testing was used to evaluate the influence of PBSA content and the effect of PBSA-*g*-MAH on the toughness properties of the PHBV/PBSA films. The film samples were also evaluated in both MD and TD, and their response was recorded after 15 days of aging. The parameters derived from these tests are outlined in Figure 7, whereas Figure 8 presents the specimens tested. The neat PHBV film exhibited brittle behavior with crack deflection in MD, indicating low toughness. The neat PBSA, on the contrary, showed extensive ductile behavior with high toughness, in both testing directions. The addition of 15 %wt PBSA to PHBV, with or without PBSA-*g*-MAH, did not result in significant differences in tear strength compared to the unblended PHBV film. However, these 85:15 (*wt*/*wt*) blends did not exhibit crack deflection and plastic deformation was observed along the crack sides. On the other hand, blend films with 30 %wt PBSA showed a decrease in tear strength compared to the neat PHBV film. Indeed, the 70:30 (*wt*/*wt*) films also exhibited crack deflection and brittle behavior, confirming the lower mechanical performance attained for this blend composition.

**Figure 9:**
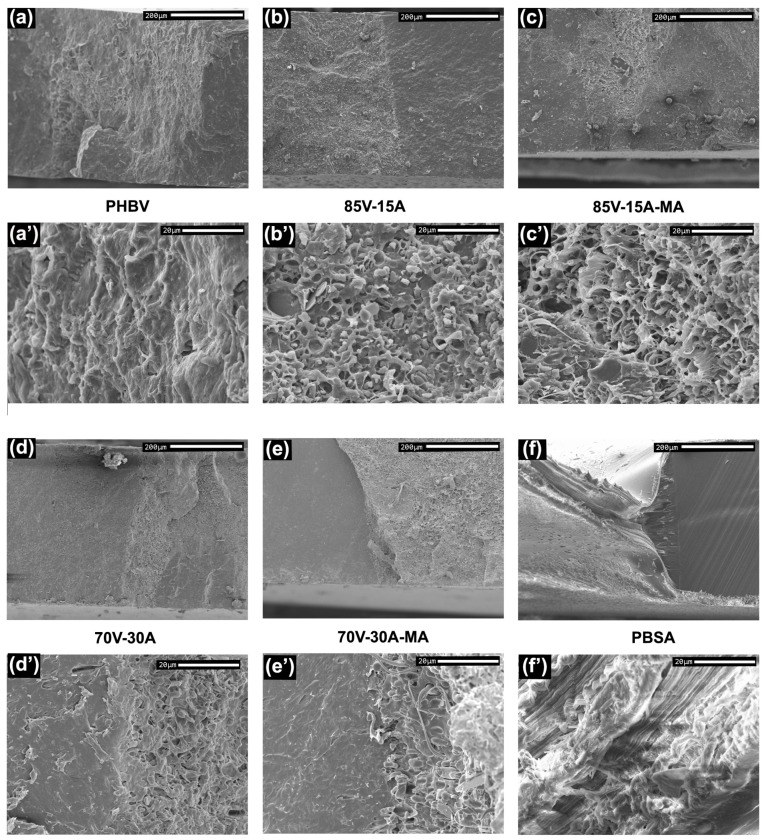
Scanning electron microscopy (SEM) micrographs of the fracture surfaces of (**a**,**a′**) neat poly(3-hydroxybutyrate-*co*-3-hydroxyvalerate) (PHBV); (**b**,**b′**) 85V-15A; (**c**,**c′**) 85V-15A-MA; (**d**,**d′**) 70V-30A; (**e**,**e′**) 70V-30A-MA; (**f**,**f′**) neat poly(butylene succinate-*co*-adipate) (PBSA). Images taken at 200× and 1500× magnifications.

In order to achieve a better understanding of the fracture process of the specimens, DDENT samples were tested in tension loading. This analysis reveals different fracture morphologies and the role of soft PBSA particles in the PHBV matrix. After loading the specimens, SEM micrographs were taken on the fracture surfaces to observe differences in the morphology during fracturing. These micrographs are presented at two magnifications (200× and 1500×) in Figure 9. For example, Figure 9b represents the fracture of 85V-15 A near the notch edge at 200×, and Figure 9b’ shows a magnification of the fractured zone at 1500×, where the deformed second phase can be observed. The neat PHBV film exhibited a brittle fracture with unstable crack propagation and deviation. On the other hand, PBSA showed very ductile behavior with post-yielding stable crack propagation. Regarding the blends, whilst the overall behavior of 85V-15A and 70V-30A was that of a brittle material, these blend films showed some local plastic deformation and stable crack growth during the fracture (see details in Figure 9b’,c’). When PBSA-*g*-MAH was used to compatibilize the blend, the fracture behavior did not show significant variations. However, the micrographs revealed that the deformation of the secondary-phase domains was enhanced with respect to their respective uncompatibilized blends.

### 3.4. Barrier Properties

Table 5 shows the values of WVP, LP, and OP of the films of PHBV, PBSA, and their blends, all measured at 25 °C and 53% RH. The barrier performance in relation to vapors and oxygen is, in fact, the main interest for food packaging applications. In particular, the water vapor barrier is of great importance to avoid foods’ physical and chemical deterioration related to changes in the moisture content [56].

It can be observed that the water vapor barrier of the PHBV film was 1.87 × 10^−15^ kg·m/m^2^·Pa·s, which was one order of magnitude higher than that of PBSA, that is, 2.79 × 10^−14^ kg·m/m^2^·Pa·s. These values are similar to those reported earlier for both biopolymers [57,58], which highlights the good water vapor barrier performance of PHBV mainly due to its high crystallinity. In a food packaging context, the water vapor barrier of PHBV is in the range of that of polyethylene terephthalate (PET) (2.30 × 10^−15^ kg·m/m^2^·Pa·s) [59], whereas the barrier of PBSA is closer to that of other biodegradable copolyesters, such as PBAT (3.31 × 10^−14^ kg·m/m^2^·Pa·s) [57]. In relation to the biopolyester blends, these films presented intermediate permeability values, in the 0.85–1.25 × 10^−15^ kg·m/m^2^·Pa·s range, increasing with the PBSA content. Interestingly, for both blend films, the lowest values were attained in the case of the compatibilized samples with PBSA-*g*-MAH. This improvement can be ascribed to the higher miscibility attained in the blends with the compatibilizer, which offer benefits for the preservation of moisture-sensitive products.

All the film samples presented a similar and relatively low performance in terms of the D-limonene barrier. This parameter is measured to predict the aroma permeability of a packaging material. Both polyester films showed values of LP of 1.03 × 10^−14^ kg·m/m^2^·Pa·s and 5.35 × 10^−14^ kg·m/m^2^·Pa·s for PHBV and PBSA, respectively. In this regard, one should consider that D-limonene is a strong plasticizer for polyesters. Indeed, it has been reported that neat PHBV films attained a D-limonene uptake of 12.7 wt% [60]. As in the case of water, the blend films provided aroma permeability values in between PHBV and PBSA, ranging from 1.97 to 2.69 × 10^−14^ kg·m/m^2^·Pa·s and also increasing with the PBSA content. Moreover, slightly lower values were attained in the case of the compatibilized blends with PBSA-*g*-MAH. These results frame the aroma barrier performance of the here-developed blends in the range of other biopolymers, such as PLA (3.30 × 10^−15^ kg·m/m^2^·Pa·s) and PBAT (7.26 × 10^−14^ kg·m/m^2^·Pa·s) [57], but still at least one order of magnitude higher than current commercial multilayers based on petrochemical polymers [61]. Thus, similar to other polyesters, these films should not be intended to preserve food rich in aromatics, such as vegetables, herbs, and spices.

In the case of the oxygen barrier, one can see that the neat PHBV film exhibited a low OP value of 2.16 × 10^−19^ m^3^·m/m^2^·s·Pa at 25 °C and 53% RH, which agrees with our previous results [57]. In contrast, the PBSA film exhibited notably higher permeability to oxygen, with a value 7.96 × 10^−18^ m^3^·m/m^2^·s·Pa. This value is in the range of that reported previously for a PBS/PBSA blend film, that is, 1.27 × 10^−18^ m^3^·m/m^2^·s·Pa [58], and also close to that of other biodegradable copolyesters, such as PBAT (9.14 × 10^−18^ m^3^·m/m^2^·s·Pa) [57]. In relation to the PHBV/PBSA blends, the film samples showed intermediate values, ranging from 5.96 to 7.99 × 10^−19^ m^3^·m/m^2^·s·Pa. Like in the case of vapor permeabilities, the higher the PBSA content in the blend, the higher the permeability to oxygen. Furthermore, the compatibilized blends with PBSA-*g*-MAH also presented lower values than their corresponding non-compatibilized ones. This improvement further confirms the better dispersion of PBSA in PHBV in the films compatibilized by PBSA-*g*-MAH, which successfully reduced oxygen diffusion. Furthermore, the resultant barrier performance against oxygen puts these PHBV/PBSA blends in the range of medium-oxygen-barrier materials. For instance, the OP values are relatively close to those of PET films measured at 23 °C and 0% RH (3.27 × 10^−19^ m^3^·m/m^2^·s·Pa) and 75% RH (4.26 × 10^−19^ m^3^·m/m^2^·s·Pa) [62]. Moreover, these biopolyester blends outperform PLA (2.22 × 10^−18^ m^3^·m/m^2^·s·Pa) at 25 °C and 60% RH [57]. Thus, the here-developed biopolyester blend films can be applied to preserve foodstuffs that are sensitive to oxidation processes (e.g., meat, fish, or high-lipid-content products).

## 4. Discussion

The addition of PBSA-*g*-MAH to PHBV/PBSA blends during cast-film extrusion resulted in films with slightly higher thermal resistance, improved mechanical properties, and lower permeability (hence higher barrier) to water and aroma vapors and oxygen when compared to the same biopolyester blend films without the reactive agent. The morphological analysis revealed that two factors can provide this physical improvement, namely, compatibilization between the phases [53,54,55] and size of the dispersed domains [20,44,45]. However, the enhancement was mainly observed for the PHBV/PBSA 85:15 (*wt*/*wt*) blend. In the case of the 70:30 (*wt*/*wt*) blend, it was seen that the size of the PBSA droplets was much larger. This result suggests that the physical properties of PHBV/PBSA blends are controlled by the domain size of the particles and the PBSA-*g*-MAH effect was restricted for blend compositions with lower amounts of the secondary phase.

These findings align with previous studies. Feijoo et al. [18] reported similar results for PHBV/PBSA blends with a chain extender, noting that the addition of PBSA enhanced the properties of neat PHBV. Furthermore, incorporating the chain extender improved compatibility between the two polymers, leading to enhanced properties, especially at higher PHBV concentrations. Rojas-Lema et al. [33] found that adding MAH as an additive enhanced the interfacial interaction between PBS and pistachio filler, resulting in improved mechanical and thermomechanical rigidity and hardness.

**Figure 10:**
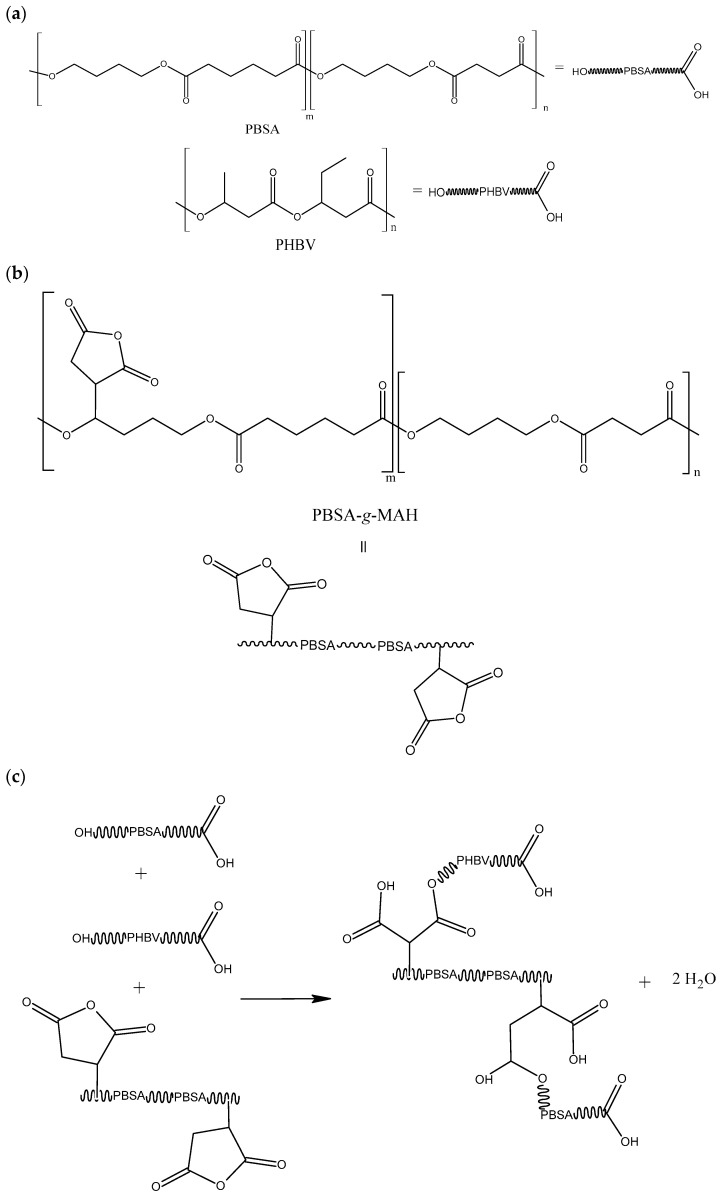
Schema to illustrate the reaction of the terminal groups of PHBV and PBSA with PBSA-*g*-MAH. The drawings depict (**a**) the terminal groups of PHBV and PBSA, (**b**) the structure of PBSA-g-MAH, and (**c**) the proposed reaction between them.

The compatibilization achieved in the PHBV/PBSA blends by PBSA-*g*-MAH can be ascribed to a REX process by which different biopolymers can be linked by covalent bonds [57,63,64]. A scheme of the potential reaction mechanism during REX is proposed in Figure 10. On the one hand, PBSA-*g*-MAH is highly soluble in PBSA, forming a single secondary phase to be dispersed within the PHBV matrix. On the other, some of the multiple MAH groups present in PBSA-*g*-MAH can react with the hydroxyl (–OH) end groups of both PHBV and PBSA, which are present in the acid or alcohol terminal groups of the biopolyesters, as fully described in our previous research [33]. The proposed scheme suggests, as a plausible example, the ring opening and partial reaction of MAH groups of PBSA-*g*-MAH with terminal –OH groups of both biopolyesters, forming a single ester function and a free carboxylic group. Alternatively, anhydride can also completely react to form di-ester structures with the same different biopolyesters [65]. Thus, the PHBV chains are connected to PBSA chains through newly formed ester groups, which at the same time, are connected to PBSA chains.

## 5. Conclusions

The REX process by means of PBSA-*g*-MAH allows us to develop PHBV/PBSA blends with improved thermal resistance, mechanical performance, and barrier properties compared to the same uncompatibilized blend. In particular, the reactive compatibilization slightly delayed the onset of thermal degradation, promoted the development of plastic deformation during fracture, and reduced the vapor and gas permeability of the biopolyester blend films. However, these improvements were only achieved for PBSA contents of 15 wt%, and these were hardly noticeable in the case of the 70-30 (*wt*/*wt*) blend. The effect’s dependence on the blend composition was ascribed to the size of the dispersed domains. Therefore, the results of this study suggest that PBSA-*g*-MAH has the potential to act as a compatibilizer between PHBV and PBSA, and the resultant blends can be of interest as rigid packaging materials to preserve moisture- and oxygen-sensitive products. Future studies will deal with the processability of the biopolyester blends to create packaging structures and further analyze their migration into food simulants and use to preserve foodstuffs.

## Figures and Tables

**Table 1:** List of films of poly(3-hydroxybutyrate-*co*-3-hydroxyvalerate) (PHBV)/poly(butylene succinate-*co*-adipate) (PBSA) blends compatibilized by poly(butylene succinate-*co*-adipate) grafted with maleic anhydride (PBSA-*g*-MAH).

Sample	PHBV (wt%)	PBSA (wt%)	PBSA-*g*-MAH (phr)
PHBV	100	-	-
85V-15A	85	15	-
85V-15A-MA	85	15	3
70V-30A	70	30	-
70V-30A-MA	70	30	3
PBSA	-	100	-

**Table 2:** Particle size distribution of the poly(butylene succinate-*co*-adipate) droplets in the blends.

	85V-15A	85V-15A-MA	70V-30A	70V-30A-MA
d_10_ (µm)	0.65	0.55	0.65	0.50
d_50_ (µm)	1.40	1.65	2.15	1.95
d_90_ (µm)	2.45	3.70	>4	3.90
Average size (µm)	1.47	1.90	2.42	2.13

**Table 3:** Onset degradation temperature measured at 5% of weight loss (T_5%_) and degradation temperature (T_d_) of neat poly(3-hydroxybutyrate-*co*-3-hydroxyvalerate) (PHBV), poly(butylene succinate-*co*-adipate) (PBSA), and their blends.

Materials	T_5%_ °C	T_d (PHBV)_ °C	T_d (PBSA)_ °C
PHBV	279	299	-
85V-15A	279	295	393
85V-15A-MA	282	298	398
70V-30A	283	298	399
70V-30A-MA	283	286	399
PBSA	357	-	407

**Table 4:** Crystallization enthalpy (ΔH_c_) and temperature (T_c_), melting enthalpy (ΔH_m_) and temperature (T_m_), and percentage of crystallinity (X_c_) of neat poly(3-hydroxybutyrate-*co*-3-hydroxyvalerate) (PHBV), poly(butylene succinate-*co*-adipate) (PBSA), and their blends.

	PHBV							PBSA								
	First Heating		Cooling		Second Heating			First Heating		Cooling			Second Heating			
Film	ΔH_m1_ (J/g)	T_m1_ (°C)	ΔH_c1_ (J/g)	T_c1_ (°C)	ΔH_m1_ (J/g)	T_m1_ (°C)	Χ_c1_ (%)	ΔH_m2_ (J/g)	T_m2_ (°C)	ΔH_c2_ (J/g)	T_c2.1_ (°C)	T_c2.2_ (°C)	ΔH_m2_ (J/g)	T_m2.1_ (°C)	T_m2.2_ (°C)	Χ_c2_ (%)
PHBV	85	169	90	120	91	171	62	-	-	-	-	-	-	-	-	-
85V-15A	64	168	72	117	74	170	60	6	86	3	39	54	5	-	87	27
85V-15A-MA	71	169	72	118	75	170	62	6	86	4	40	54	5	-	87	27
70V-30A	62	173	60	117	63	170	62	12	87	5	41	55	11	79	87	26
70V-30A-MA	61	172	59	116	63	169	63	12	87	4	41	53	11	78	87	28
PBSA	-	-	-	-	-	-	-	41	89	41	-	61	41	83	89	31

**Table 5:** Permeability to water vapor (WVP), D-limonene (LP), and oxygen (OP) of neat poly(3-hydroxybutyrate-*co*-3-hydroxyvalerate) (PHBV), poly(butylene succinate-*co*-adipate) (PBSA), and their blends.

Sample	WPV × 10^14^ (kg·m/Pa·s·m^2^)	LP × 10^14^ (kg·m/Pa·s·m^2^)	OP × 10^19^ (m^3^·m/Pa·s·m^2^)
PHBV	0.19 ± 0.04	1.03 ± 0.70	2.16 ± 0.08
85V-15A	1.15 ± 0.22	2.29 ± 0.28	6.79 ± 0.37
85V-15A-MA	0.86 ± 0.15	1.97 ± 0.17	5.96 ± 0.15
70V-30A	1.24 ± 0.21	2.69 ± 0.23	7.99 ± 0.51
70V-30A-MA	1.09 ± 0.23	2.38 ± 0.12	6.75 ± 0.13
PBSA	2.79 ± 0.17	5.35 ± 0.22	79.62 ± 1.94

## Data Availability

Data are contained within the article.
